# COVID-19 Thrombosis: Bridging the Old and New Concepts

**DOI:** 10.21315/mjms2021.28.1.2

**Published:** 2021-02-24

**Authors:** Ratika Gupta, Omkar Kalidasrao Choudhari, Hemendra Mishra, Umesh Chandra Ojha

**Affiliations:** 1 Radiation Oncology Department, Vardhman Mahavir Medical College and Safdarjung Hospital, New Delhi, India; 2 Department of Biochemistry, Vardhman Mahavir Medical College and Safdarjung Hospital, New Delhi, India; 3 Employee State Insurance Corporation Post Graduate Institute of Medical Science and Research, New Delhi, India

**Keywords:** thrombosis, COVID-19, hypercoagulable, inflammation, cytokines, endothelial dysfunction

## Abstract

The coronavirus disease 2019 (COVID-19) pandemic is an evolving condition in the absence of established treatment and vaccines. The few autopsy studies on COVID-19 patients suggested the presence of pulmonary microvascular thrombosis. Hence, it is imperative to understand the pathobiology of thrombus formation and speculate the therapeutic goals in combating COVID-19. This paper focuses on a holistic approach by integrating the previous concepts and current concepts of thrombosis to better understand the pathogenesis of thrombosis.

## Introduction

The coronavirus disease 2019 (COVID-19) pandemic has emerged as the major global health concern for humans; it has caused more than 400,000 deaths worldwide (1). In addition, none of the patients have prior immunity to this viral infection and, as of now, no specific proven treatment and vaccine are available. Clinical presentation in patients varies from asymptomatic to mildly symptomatic to being critically ill, which may be due to various pathophysiological changes that may also cause mortality. Recently, thromboinflammation, considered as an interaction between inflammation and coagulation, has been documented as the probable cause of microthrombi formation. It manifests as an acute lung injury and leads to hypoxemic respiratory failure, where high oxygen flow does not improve patients’ oxygen saturation due to mismatch between ventilation and perfusion. This respiratory failure has been described as the most common cause for deaths due to COVID-19 (2–5). Clinical signs of deep vein thrombosis and pulmonary embolism, confirmed by imaging or autopsy, have also been documented in COVID-19 patients (6). Their autopsy findings have demonstrated diffuse alveolar damage, pulmonary microvascular thrombosis in an environment of marked inflammatory changes, small vessel thrombosis in multiple organs and pulmonary emboli (7). As reported in the literature, 20% of patients under extracorporeal membrane oxygenation had pulmonary embolism (8). Hence, it is imperative to understand the pathogenesis of thrombus formation. This review paper aims to understand the mechanism of COVID-19 thrombosis and attempts to build a bridge between our past knowledge of thrombosis and new concepts learnt during COVID-19 pandemic.

## Role of Platelets in Bringing Hyper-Coagulable State

Platelets have long been involved in the manifestation of viral illnesses. Thrombocytopenia is seen in adenovirus, coxsackie virus, hepatitis B and C viruses, and filovirus (9, 10). Several mechanisms, including early clearance of activated platelets or the virus and platelets interaction or the direct infection of megakaryocytes have been suggested for thrombocytopenia (11). The roles of platelets in innate immunity include secreting adhesion molecules for the attachment of leucocytes at the site of injury via intercellular adhesion molecule-2 and β2 integrin interaction, and triggering the inflammatory cascade (12, 13). Studies on COVID-19 patients have showed the presence of thrombocytopenia and its association with poor prognosis (14). The pathogenesis of thrombus formation with thrombocytopenia is probably due to the activation of platelets by complement system. This results in more thrombus formation via the generation of procoagulant micro-particles and insertion of C5b-9 in lytic amount like in malignancies leading to cell lysis or in the absence of fluid phase or membrane bound platelet integrity complement regulators (15, 16). Secondly, the cascade of uncontrolled cytokines’ multiplication induced by activated platelets and complement system leads to subsequent complications like disseminated intravascular coagulation (DIC) (17). In a study by Tang et al. (18), 71.4% of non-survivors and 0.6% of survivors of COVID-19 met the criteria of the International Society on Thrombosis and Haemostasis for DIC with enhanced fibrinolysis rather than suppressed fibrinolysis, caused by infectious diseases (where fibrin degradation product [FDP] and D-dimer levels are mildly elevated and fibrinogen is not decreased) (19).

## Endothelial Dysfunction

The endothelial cells generally express or secrete an assortment of endogenous anticoagulants, which act at specific sites along the coagulation cascade to inhibit coagulation. However, this endothelial lining may get disrupted due to endothelial injury, vascular leakage and down-regulation because of elevated levels of inflammatory cytokines in infected patients (20). The pathophysiology of the virus suggests that it accesses host cells via the protein angiotensin-converting enzyme 2, which is highly expressed in lung alveolar cells, cardiac myocytes and primarily in vascular endothelium. It is assumed that the COVID-19 virus targets the endothelium. This assumption can be proved by the fact that endothelial dysfunction is known to be a key determinant in hypertension, thrombosis and DIC, as demonstrated in several clinical findings of COVID-19 patients (21, 22).

In lungs, similar phenomenon is observed as infection with COVID-19 initiates alveolar injury, resulting in inflammatory response that includes production of inflammatory cytokines (such as IL-1β, IL-2, IL-6, IL-8, TNF-α and IL-10), which lead to more injury to tissues and the capillary endothelium. Although IL-10 is considered an anti-inflammatory cytokine, its paradoxical behaviour was observed in COVID-19 and several other conditions (23, 24). The usual thrombo-protective state of the vascular endothelial cells is, thus, disrupted, accentuated by the procoagulant effectors derived from inflammation (including cytokines, neutrophil extracellular traps and polyphosphates). These pathophysiologic changes lead to the development of microvascular thrombosis. This, in turn, leads to vascular occlusion, followed by progressive hypoxemic respiratory failure and acute respiratory disease syndrome (ARDS), which is the major cause of mortality in patients presenting with COVID-19 (25). COVID-19 can cause severe life-threatening medical conditions such as systemic inflammatory response syndrome and ARDS that may involve multiple organs and shock (26). Many pro-inflammatory cytokines are known to trigger the coagulation system, but Zhou et al. (4) showed that the rise in IL-6 cytokine was much later than that in D-dimer. This suggests that the high D-dimer levels observed in COVID-19 patients are secondary to systemic inflammation and reflect true thrombotic disease. This increased thrombotic state can manifest as clinical venous thromboembolic (VTE) events and develop into DIC, which may evolve into consumptive coagulopathy (27).

## Venous Stasis

Critically ill patients, including those suffering from pneumonia, are more susceptible to developing VTE. In addition, this VTE development is further enhanced by several factors: i) the prolonged mechanical ventilation and its attendant hemodynamic effects; ii) the presence of central venous catheters; and iii) prolonged immobilisation. The presence of comorbidities like obesity further increases the risk of venous stasis. In several findings, the overall rate of VTE in patients was found to range from 25% to 69% (8, 28).

VTE can be overlooked due to various reasons: i) respiratory symptoms attributed to pneumonia or ARDS; ii) thrombosis not well identified on chest radiography; iii) inability to perform computerised tomography scans because of practical issues and iv) the assumption that prophylactic anticoagulation might be a saviour in prevention of thrombosis in all cases. However, good clinical assessment coupled with relevant laboratory testing can raise the suspicion of VTE for early intervention (29). The most important diagnostic test is D-dimer assay, showing D-dimer levels as markers of disease severity and are predictive of mortality (30). D-dimer cut-off of 1.5 μg/mL has been used for predicting VTE (31). In case of patients with coronavirus-associated pneumonia, the non-survivors revealed significantly higher D-dimer and FDP levels, and longer prothrombin time (PT) compared to survivors. These abnormal coagulation results could help guide therapy with anticoagulants and evaluate prognosis (18). Spontaneous prolongation of PT > 3 sec and activated partial thromboplastin time > 5 sec have also been demonstrated as independent predictors of thrombotic complications (32).

Anticoagulant therapy remains the mainstay of treatment for VTE. Low molecular weight heparin (LMWH) is preferred for VTE treatment over unfractionated heparin (UFH) due to its ease of use, non-requirement of laboratory monitoring, and familiarity among the spectra of doctors with varying experiences. Though the role of LMWH or UFH in prophylaxis for all COVID-19 positive patients is not yet proven, they can be administered to cases of high suspicion of pulmonary embolism and when imaging is infeasible (33). Moreover, apart from its anticoagulant effect, heparin has shown anti-inflammatory functions, endothelial protection and viral inhibition that may be helpful in fighting against COVID-19 (34).

Venous stasis, endothelial dysfunction, platelets and complement system derived hypercoagulable state to be the common pathophysiology in patients infected by COVID-19, leading to complicated conditions like VTE, DIC and pulmonary embolism. The COVID-19 coagulopathy manifests with enhanced fibrinolysis as usually seen in leukaemia with severe coagulation and fibrinolysis activation in contrast to sepsis-induced DIC where fibrinolytic activity is minimal. Early laboratory and radiological investigations were suggested in suspected patients and anticoagulant therapy should be started.

**Figure 1:**
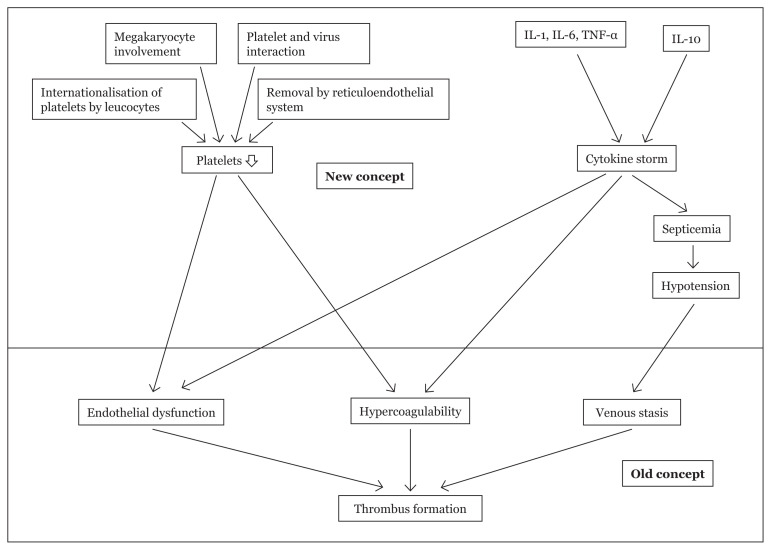
Connecting new concepts of thrombus formation in COVID-19 with old concepts

Rudolf Virchow (1856), a German pathologist postulated a triad for thrombosis comprising of hypercoagulability, venous stasis, and endothelial dysfunction (35). This triad built the fundamental pathobiology behind thrombus formation, which is true in the current scenario of COVID-19 manifestations as well (Figure 1). The endothelium plays a pivotal role in cytokine storm, and more studies are required to understand its versatile role in the inflammation probably by endothelium modulators.

## Conclusion

In view of hypercoagulable state in COVID-19, prophylactic LMWH should be initiated in clinically severe disease. The endothelial modulators may play a role in management of COVID-19 infection and prophylactic ambulation in all patients of COVID-19 is needed to avoid venous stasis.
